# Does chromoanagenesis play a role in the origin of B chromosomes?

**DOI:** 10.1038/s41437-025-00758-w

**Published:** 2025-04-19

**Authors:** Andreas Houben, Jörg Fuchs, Ali Mohammad Banaei-Moghaddam, Jianyong Chen, Gihwan Kim, Taoran Liu

**Affiliations:** 1 Leibniz Institute of Plant Genetics and Crop Plant Research (IPK) Gatersleben, 06466 Seeland, Germany; 2 Laboratory of Genomics and Epigenomics (LGE), Department of Biochemistry, Institute of Biochemistry and Biophysics (IBB), University of Tehran, Tehran, Iran

**Keywords:** Cytogenetics, Evolution

## Abstract

B chromosomes (Bs) exist in addition to the standard (A) chromosomes in a wide range of species. The process underlying their origin is still unclear. We propose pathways of intra- and interspecific origin of B chromosomes based on known mechanisms of chromosome evolution and available knowledge of their sequence composition in different species. We speculate that a mitotic or meiotic segregation error of one or more A chromosomes initiates, via chromoanagenesis, the formation of a proto-B chromosome. In the second step, proto-B chromosomes accumulate A chromosome- and organelle-derived sequences over time, most likely via DNA double-strand break (DSB) mis-repair. Consequently, the original structure of the early stage proto-B chromosomes becomes masked by continuous sequence incorporation. The similarity between A chromosome sequences integrated into B chromosomes and the original sequences on the donor chromosomes decreases over time if there is no selection pressure on these sequences on B chromosomes. However, besides chromoanagenesis, also other mechanisms leading to the formation of B chromosomes might exist.

The B chromosome (B) is a supernumerary part of the genome of several fungi, plants and animals. Depending on the species, Bs may vary in size and number, but share specific characteristics that make them unique and distinguishable from other types of chromosome number polymorphisms, like aneuploidy (reviewed in Ferree et al. 2024). B chromosomes and standard chromosomes (As) use the same cellular machinery to replicate and transmit across generations. Most Bs confer no detectable impact on the carrier organism at low numbers, but increased numbers can result in phenotypic aberrations and reduced fertility (Bougourd and Jones 1997). They are, therefore, considered as parasitic or selfish. However, in some species, Bs became an essential part of the genome, turning into new sex chromosomes, germline-restricted chromosomes (GRCs) or influencing pathogenicity (reviewed in Johnson Pokorna and Reifova 2021). In contrast to B chromosomes, studies have shown that A chromosome aneuploidy induces distinct phenotypic effects (McClintock 1929).

Despite the fact that the first B chromosome was reported more than 100 years ago (Wilson 1907), the process underlying their origin is still unclear. B chromosomes most likely developed differently in various species but probably share certain evolutionary processes. A widely accepted view is that most B chromosomes are derived from the A chromosome complement of the B chromosome-carrying species. Some evidence also suggests an interspecific origin (Jones and Houben 2003). In this case, Bs spontaneously generated in response to the new genomic conditions after interspecific hybridization. The involvement of sex chromosomes has also been argued for their origin in some animals (see for examples Camacho et al. 2000). However, despite the high number of species with Bs, their formation is probably a rare event because in many species, only one or few variants of B chromosomes exist (e.g. *Secale* (Chen et al. 2024; Jones and Puertas 1993; Marques et al. 2013), *Brachyscome* (Houben et al. 1999)).

This long-lasting question now become addressable because even complex and repeat-rich chromosomes, such as Bs, can be sequence-assembled and analyzed by advanced technologies. Comprehensive sequence analysis of plant B chromosomes was initiated using a combination of chromosome sorting and next-generation 454 sequencing (Martis et al. 2012). The most surprising finding was that the rye B carries genic sequences derived from the entire rye A chromosome complement. In addition, compared to As, the B was found to accumulate larger amounts of organelle-derived DNA. Thus, the B chromosome acts like a “genomic sponge”, which collects nuclear-, plastid- and mitochondrion-derived DNA (Chen et al. 2024; Martis et al. 2012) (Fig. 1). Besides pseudogenes, the rye B contains A chromosome-derived functional genes (Banaei-Moghaddam et al. 2013; Ma et al. 2017; Ma et al. 2021).

**Fig. 1:**
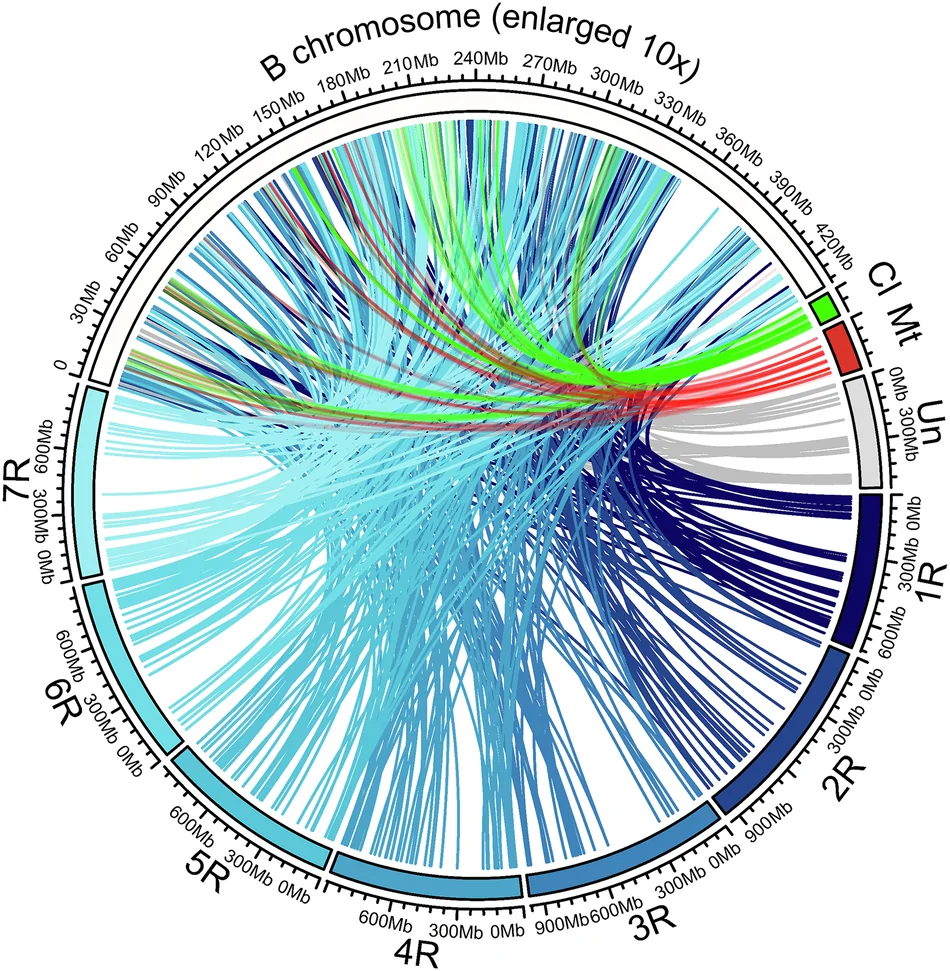
The rye B chromosome possesses a complex, mosaic-like sequence composition. Circos plot shows the relationship between B-located genes and high-confidence genes of the A chromosome and the organellar genome of rye (Chen et al. 2024). Rye B chromosome was 10 fold enlarged (white), 1R - 7R: rye A chromosomes (different shades of blue). The rye A chromosome sequence was determined by (Rabanus-Wallace et al. 2021). Un: unassigned scaffold of the A chromosomes (gray). Cl Chloroplast DNA (green). Mt Mitochondrial DNA (red). Cl and Mt were 200 and 100, respectively.

A complex sequence composition that can not be explained by just a few chromosome translocation events was also reported for the Bs of the goatgrass *Aegilops speltoides* (Ruban et al. 2020), maize (Blavet et al. 2021; Liu et al. 2025), saltgrass (Nawaz et al. 2024), common reed (Cui et al. 2025; Wang et al. 2024), hornworths (Schafran et al. 2025), red fox and raccoon dogs (Makunin et al. 2018), for the B chromosome-like germline-restricted chromosomes (GRCs) of songbirds (Kinsella et al. 2019; Schlebusch et al. 2023) and gnats (Hodson et al. 2022), the paternal sex ratio (PSR) chromosome, a B chromosome in the jewel wasp *Nasonia* (Dalla Benetta et al. 2020), the “B-sex” chromosome in the cavefish, *Astyanax mexicanus* (Imarazene et al. 2021), and the B chromosome-like accessory chromosomes in fungi (Habig and Stukenbrock 2020; Langner et al. 2021).

## How does the sequence complexity of B chromosomes evolve?

Based on our and other studies, we suggest a multi-step process for the de novo origin and evolution of sequence-complex Bs. Sequence-complex Bs refer to chromosomes composed of essential chromosome components, such as centromere and telomeres and also a substantial amount of noncoding/coding sequences of multiple A chromosomes and organell-derived DNA. We propose that a chromoanagenesis-like process might initiated the formation of proto-Bs. Chromoanagenesis (from the Greek *chromo* for chromosome and *anagenesis* for rebirth) is a term encompassing all types of catastrophic chromosomal rearrangements occurring from a single cellular event, including chromothripsis, a process initially described as a signature of cancer. Recent studies have shown that chromoanagenesis can also happen in human germline cells and during early embryonic development (Zepeda-Mendoza and Morton 2019).

A chromoanagenesis-derived chromosome results from complex chromosomal rearrangements after a catastrophic event occurring within a short time window and involving multiple double-strand breaks (DSBs) within one or more chromosomes. Mechanistically, chromoanagenesis originates from chromosome segregation errors, resulting in the formation of micronuclei. Due to intricate defects associated with the nuclear envelope, micronuclei are susceptible to DNA damage and chromosome fragmentation (Hatch et al. 2013). Some of these broken segments are then reassembled in random order and orientation, leading to the formation of derivative chromosomes with unique structures, including copy number changes within the newly formed chromosome (Pellestor et al. 2022; Stephens et al. 2011).

Chromoanagenesis has also been discovered in non-cancer cells of various species, including plants (Guo et al. 2023; Pellestor and Gatinois 2020). Studies suggest that catastrophic chromosome rearrangements might even have contributed to the evolution of A chromosomes in some species, e.g. of *Camelina* (Mandakova et al. 2019) and the ‘zebra’ chromosome z5A identified in derivatives of an *Elymus trachycaulus* x *Triticum aestivum* hybrid (Jiang and Gill 1993). Chromosome shattering and reshuffling of chromosome fragments are also involved in the formation of mammalian B chromosome-like complex small supernumerary marker chromosomes (SMCs) (Cernohorska et al. 2023; Liehr et al. 2008; Weber et al. 2022). In these cases, the chromosome restructuring was much less complex than in the case of e.g. cereal Bs. However, chromoanagenesis is detrimental in most instances and cells carrying the products do not survive (Mazzagatti et al. 2024).

## Intraspecific B chromosome origin

**Fig. 2:**
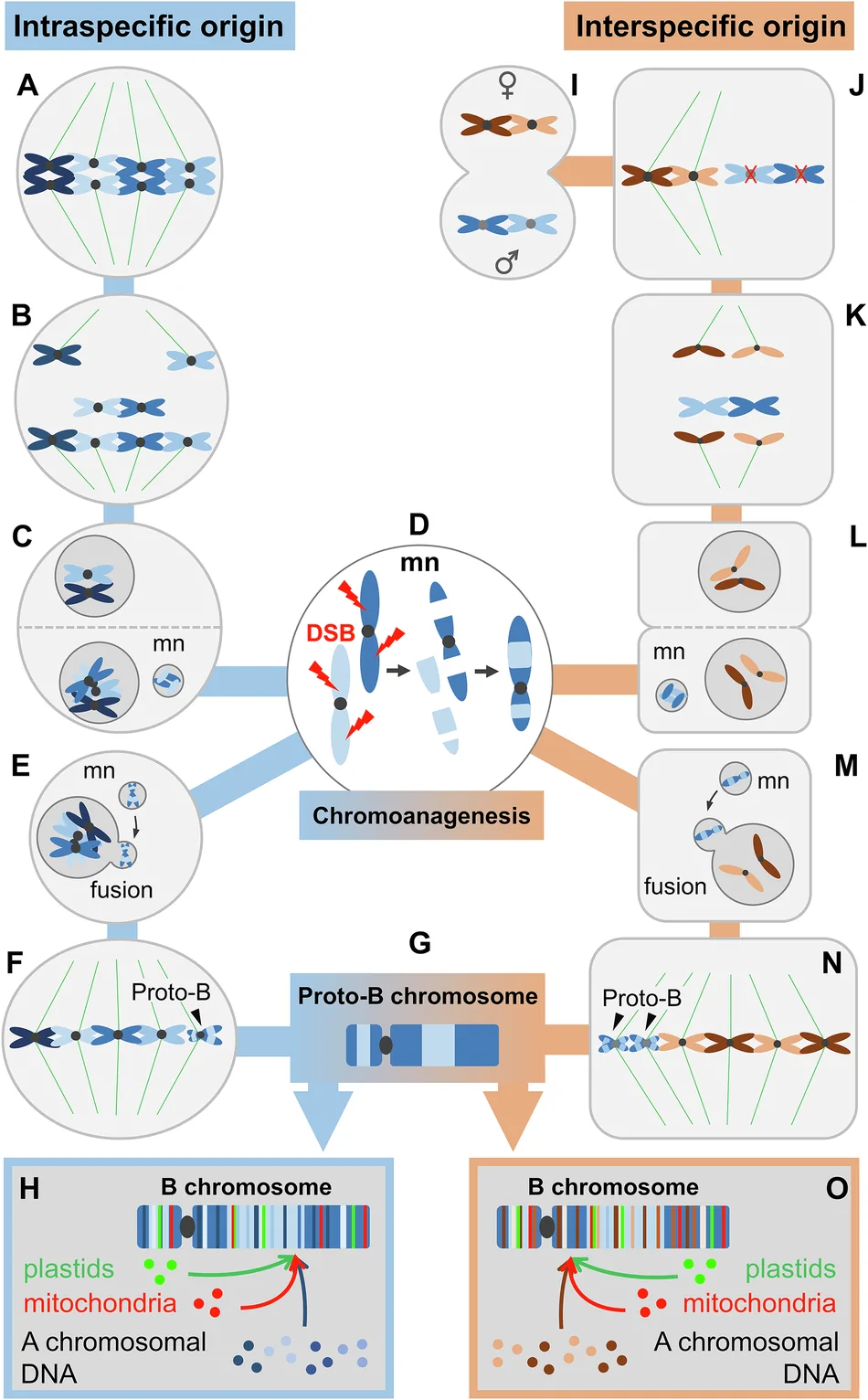
Hypothesis on the intra- and interspecific origin and evolution of a sequence complex B chromosome. **A**–**H** Intraspecific B chromosome origin. **A**, **B** The B chromosome formation is initiated by the missegregation of a few A chromosomes at meiosis I. **B**, **C** Lagging As undergo micronucleation and fragmentation. **D** The chromoanagenesis-like process results after the fragmentation (red arrows indicate DNA double strand breaks (DSB)) of one or several As and random fusion of A fragments in the de novo formation of a proto-B (**E**) before or after fusion of the micronucleus with a standard nucleus. Meiosis II is not shown. **F** The proto-B containing gamete results after sexual combination with a wild-type gamete in the next generation (F1) carrying a proto-B. **G** To counteract purifying selection, a B chromosome drive mechanism evolves early in the evolution of the proto B. **H** Subsequently, the proto-B chromosome collects sequences derived from all A chromosomes as well as plastids and mitochondria over time. The reduced selection pressure on the B chromosome allowed a random integration of sequences, preferably in a non-functional state, to avoid genetic imbalance. The sequence similarity between integrated sequences and the donor sequences will decrease over time. Consequently, the original structure of the proto-B chromosomes is masked by subsequently collected sequences. **I**–**O** Interspecific B chromosome origin. **I** Interspecific B chromosome formation is initiated by the hybridization of two species and the formation of an unstable hybrid genome. **J**, **K** Centromere inactivation of one parental genome will lead to the formation of lagging chromosomes. **l** Eliminated uniparental As undergo micronucleation. **D**, **M** The chromoanagenesis-like process results after the fragmentation (red arrows indicate DNA double strand breaks (DSB)) of one or several uniparentally eliminated As and random fusion of A fragments in the de novo formation of a proto-B before or after fusion of the micronucleus with a standard nucleus. Spontaneous genome doubling of the haploid somatic cell is not shown. **N** The proto-B containing gamete results after sexual combination with a wild-type gamete in the next generation (F1) carrying a proto-B. **G** To counteract purifying selection, a B chromosome drive mechanism evolves early in the evolution of the B. **O** Subsequently, the proto-B chromosome collects over time sequences derived from all As as well as plastids and mitochondria as described above (**H**). mn micronucleus, NHEJ nonhomologous end joining, Proto-B proto B chromosome.

In the case of an intraspecific B chromosome origin, we speculate that the de novo B chromosome formation could be initiated by the missegregation of one or few A chromosomes (aneuploidy) during gamete development, resulting in lagging chromosomes and micronuclei. Next, micronucleated chromosomes are shattered and reshuffled during a catastrophic event (Fig. 2A–D). Non-homologous end joining (NHEJ) of DNA breaks has been implicated in reassembling chromosome fragments generated by chromoanagenesis (reviewed in Mazzagatti et al. 2024; Pellestor et al. 2022). Such a “Frankenstein” chromosome must be “stitched” together in a way that critical components required for chromosome function, such as an active centromere and telomeres at terminal positions, are maintained.

Usually, micronuclei are eliminated; however, if they fuse with the standard nucleus and release the reassembled chromosomes into the nucleus (Tan et al. 2015), the formation of a B could be initiated. Moreover, chromosomes that move into micronuclei could experience a heritable alteration of DNA methylation patterns, that persists after their reincorporation into the primary nucleus and stimulate further epigenetic reprogramming (Agustinus et al. 2023). After a spontaneous fusion of a micronucleus and a standard nucleus, a gamete with the standard number of As and an additional proto-B evolves (Fig. 2E). The sexual combination of a proto-B-containing gamete with a wild-type gamete results in an F1 plant possessing a proto-B. Such a newly “born” proto-B does not meiotically recombine with its A chromosome progenitors anymore and could start its independent evolution, if it does not get lost in the next sexual reproduction cycle. Reshuffling of chromosome segments seems to be relatively common also in plants (reviewed in Guo et al. 2023), but A chromosome-derived proto-Bs will be tolerated over generations only if they have no or only weak gene copy number effects on the host. Figure 2F, G shows a proto-B formed after a reshuffling of fragments derived from two As. To counteract purifying selection, a drive mechanism or a fitness benefit is a prerequisite for a proto-B to evolve into a B chromosome, as simulation studies have demonstrated (Camacho et al. 1997). The occurrence of B chromosomes in certain species or populations of a given species may be explained by the presence of enabler mutations, facilitating missegregation of chromosomes, micronuclei formation and chromoanagenesis, as prerequisites for the formation of proto-B chromosomes. While this scenario implies the formation of various proto-Bs, only some of them may escape from early purifying selection, if they gain an efficient drive mechanism (Chen et al. 2022).

Subsequently, over time, the A-B chromosome synteny of the initial proto-B became masked by mutations and by random integration of further A chromosome and organelle sequences (Fig. 2H). Thus, B sequences of the initially involved A regions are less similar to their regions of origin than sequences that accumulated later during the evolution of the B chromosome (Banaei-Moghaddam et al. 2013). Alternatively, no A-B chromosome synteny is expected if the minimal proto-B chromosome only contained a centromere, flanking pericentromeric chromatin and telomeres. In the second phase, the minimal proto-B might act as a “sponge” for random A chromosome- and organelle-derived sequences. Some partial synteny between both chromosome types could be expected if such a minimal proto-B chromosome is additionally composed of scrambled fragments derived from the initial or a few other A chromosomes. However, in both cases, both sequence gain and loss are continuous and, over time would degrade the synteny.

To what extent sequence trafficking from A to B chromosomes occur during DNA double-strand break (DSB) mis-repair or via hitchhiking of genomic fragments with transposable elements, as demonstrated for non-collinear genes of Triticeae (Mascagni et al. 2021; Wicker et al. 2011) or on both routes is unknown. In addition, extrachromosomal circular DNA (eccDNA), a type of DNA that exists outside chromosomes, might contribute to the evolution of B chromosomes by integration into DSBs on Bs. This type of DNA has been detected in every eukaryotic genome tested so far (Ling et al. 2021). While the population of eccDNA may include intermediates of mobile elements or viral genomes, we refer to eccDNA as the circular molecules that are derived from the nuclear genome. They are heterogeneous in size, ranging from hundreds of base pairs to several kilobase pairs and are likely generated by DNA breakage and/or excision during biotic and abiotic stresses or via reverse transcription (Mohan et al. 2024; Zhuang et al. 2024). In plants, even B chromosome-derived eccDNA has been detected (Cohen et al. 2008). However, whether and how eccDNA contributes to the evolution of Bs remains to be tested. In human cancer cells, the reintegration of eccDNA into the nuclear genome has been demonstrated to trigger somatic genome rearrangements (Koche et al. 2020). The occurrence of organellar DNA within plant nuclear genomes (Timmis et al. 2004), could be based on eccDNA integration too. Indeed, incorporation of circular organellar DNA fragments into nuclear DNA via double-stranded break repair has been demonstrated experimentally (Wang et al. 2018; Wang et al. 2012).

Because Bs are under little or no selection pressure, mobile elements may insert, spread and amplify, as shown for the Bs of maize (Lamb et al. 2007) and rye (Klemme et al. 2013). The general absence of crossing-over between A and B chromosomes might further facilitate the accumulation of mobile elements, as proposed for Y chromosomes (Akagi et al. 2025; Charlesworth 2008; Moraga et al. 2025).

The transfer of organellar DNA to the plant nucleus is common, but much of this DNA is also rapidly lost (Timmis et al. 2004). Bioinformatic analysis indicated that NHEJ plays a role in the incorporation of both chloroplast and mitochondrial DNA during DSB repair (Hazkani-Covo and Covo 2008; Wang and Timmis 2013). Compared to A chromosomes, the B chromosomes of rye and *Ae. speltoides* accumulated more chloroplast- and mitochondrion-derived sequences (Chen et al. 2024; Martis et al. 2012; Ruban et al. 2014; Ruban et al. 2020). Possibly, because of the reduced selection pressure, insertion into Bs has fewer deleterious genetic consequences than into A chromosomes. Thus, the risk to become counter-selected is lower. All regions of the mitochondrial and chloroplast genomes are found on the rye Bs, indicating that all sequences are transferable (Martis et al. 2012). In contrast, the B chromosome of maize carries only a tiny proportion of organell-derived DNA (Blavet et al. 2021). Whether the maize B is exceptional in this regard, has to be clarified by more investigation on Bs of other species.

## Interspecific B chromosome origin

We speculate that the de novo B chromosome formation could alternatively be initiated by the missegregation of chromosomes during early embryogenesis after wide hybridization (Fig. 2I–K). During wide hybridization, two distinct genomes are combined within a single nucleus, with the organellar DNA being of maternal origin in most cases. Such novel genome combinations are often unstable and are subject to a high degree of genetic and epigenetic reorganization (Riddle and Birchler 2003). The complete elimination of one of the parental genomes is not uncommon; it has been documented in at least 74 hybrid combinations (Ishii et al. 2016). The loss of chromosomes has been ascribed to uniparental centromere inactivation (Sanei et al. 2011) and ineffective separation of sister chromatids (Ishii et al. 2010). A programmed elimination of parental chromosomes occurs also in animals (Dedukh et al. 2024; Dedukh et al. 2020; Gibeaux et al. 2018).

We propose that in hybrid embryos, uniparental chromosomes destined for elimination may lag at anaphase and form micronuclei (Fig. 2K, L). The majority of micronuclei contain only a few chromosomes (Gernand et al. 2005). Depending on the species combination, complete elimination of one parental genome occurs in a stepwise manner within a few days or weeks (Gernand et al. 2006; Gernand et al. 2005). In the wheat x pearl millet combination, the centromere regions of pearl millet chromosomes are eliminated last (Gernand et al. 2005). Such a centric fragment could provide an ideal prerequisite for the de novo formation of a B chromosome. A similar scenario was assumed for *Coix* (Sapre and Deshpande 1987), where a B chromosome was likely generated spontaneously as a result of the crossing of two species. A reanalysis of *Coix gigantea* x *C. aquatica* with advanced genomic tools could result in more profound insights into how a B chromosome could arise after wide hybridization. Such a B should reveal many sequences derived from the eliminated genome.

As a consequence of micronucleation, a chromoanagenesis-like process initiates the formation of a proto-B (Fig. 2D). After a spontaneous fusion of a micronucleus containing a proto-B chromosome and a standard nucleus, a cell with the haploid number of maternally derived chromosomes and an additional proto-B evolves of mainly paternal origin during early embryogenesis (Fig. 2M). To ensure fertile plants, a spontaneous genome doubling must occur before the onset of meiosis. Finally, like in the case of an intraspecific B chromosome origin, the proto-B chromosome accumulates coding, non-coding A and organelle sequences over time (Fig. 2O).

In summary, heavily restructured chromosomes might evolve frequently across species, but most are eliminated early, and only rarely do such chromosomes develop into a B chromosome. The proposed mechanisms offer alternative explanations for the formation of complex B chromosomes. Of course, it can not be ruled out that additional pathways of B chromosome formation exist. For instance, in *Plantago lagopus* it was shown that the origin of the B chromosome was associated with A chromosome aneuploidy, chromosome fragmentation, 5S rDNA repeat amplification, addition of telomeric repeats, and centromeric misdivision (Dhar et al. 2002). Analysis of the *Drosophila melanogaster* B chromosomes suggests that this chromosome type formed after a centromere misdivision of A chromosome 4 during meiosis (Hanlon et al. 2018).
